# Routledge Handbook of Animal Welfare, First Edition

**DOI:** 10.1017/awf.2023.82

**Published:** 2023-09-21

**Authors:** I Anna S Olsson

**Affiliations:** Laboratory Animal Science, i3S, University of Porto, Portugal

The *Routledge Handbook of Animal Welfare* is an ambitious book project. On the one hand it covers a broader range of issues, species and situations than any previous animal welfare textbook. On the other, it goes further in challenging *status quo* than is common in books of this type. The book has seven sections with a total of 36 chapters, written by an international team of authors representing a large set of disciplines. Unavoidably, a review of a book of this dimension will only scratch the surface, and it is impossible to do justice to individual chapters, but I will nevertheless attempt to give an overview of the content.

The book starts with the section, ‘Part I: Animal welfare fundamentals’, consisting of three chapters. The first, ‘The moral status of animals: biological foundations’ is arguably more about the moral status of animals than its biological foundation, whereas the second chapter ‘Animal welfare concepts’ gives an overview of the different approaches to animal welfare. The third chapter ‘Animal welfare assessment’ gives an introduction to the concept of animal welfare as well as how to measure it.

‘Part II, Animal farming, transportation, and killing’ is the largest section, with nine chapters. It starts with a chapter that reflects critically on contemporary animal farming, its problems and the need for change. This is followed by chapters on specific types of animal farming (poultry, pigs, cattle, sheep and goats, non- and semi-domesticated terrestrial species, fish) and two final chapters on ‘Transportation’ and ‘Slaughter, euthanasia, and depopulation.’

‘Part III, Animal use for other purposes’, covers different cases of where animals are directly used to serve human interests other than in the context of farming, starting with ‘Scientific and educational animal use’. ‘Animals in entertainment’ is about animal use in tourism, circuses, sport (including combat, hunting, fishing, horse and dog racing, rodeo) and film/television. There is some overlap with ‘Hunting, fishing, and whaling’, a chapter which while generally comprehensive gives disproportionate attention to whaling. The biggest part of fishing has its own chapter, ‘Commercial fisheries.’ There is also a chapter on ‘Zoos and aquaria.’

The title of Part IV, ‘Species-specific concerns’, is somewhat misleading as it is no more species-specific than the section on farming, and a more appropriate title would be something like ‘Animals kept as companions and for interaction.’ The chapters on ‘Canines and felines’, ‘Equines’, ‘Non-domesticated terrestrial species’ and ‘Companion fish’ are about animals kept as family members or at least by private individuals, whereas ‘Marine mammals’ covers animal keeping in marine theme parks.

‘Part V, Recent and emerging issues’, includes three chapters. ‘Climate change, human-wildlife conflict, and biodiversity loss’ covers the impact (direct and indirect) of human activities on wild animals. ‘Animal welfare and human health’ introduces the concept of One Health in a comprehensive way. ‘Animal disaster management’ is about how to take animals into account when managing primarily environmental disasters.

‘Part VI Animal ethics and law’, includes two general chapters on ‘Animal ethics’ and ‘Developments in animal law’, followed by specific chapters on key animal law in different parts of the world. By including Australia, Europe, India, China, South Africa and United States, this set of chapters presents some geographic diversity but is far from comprehensive and leaves out all of one (Latin America) and most of another (Africa) continent.

‘Part VII Social change for animals’, closes the book by addressing not the situation of animals but the potential for humans in different roles to change how animals are treated. There are three chapters, on ‘Stakeholder groups and perspectives’, ‘Animal advocacy and human behaviour’, and ‘Animal welfare education and communication.’

The book goes a long way towards achieving its ambitious objective of broad coverage and challenging *status quo.* The quality of the individual chapters is somewhat variable, but the vast majority are balanced and comprehensive and will undoubtedly be very useful for readers looking for more information or deeper understanding of animal welfare in different contexts. A few chapters are written from a perspective that is explicitly personal (“I think”, “In my opinion”) or present an unjustified bias which excludes consideration of different perspectives (“[a given practice] should be banned”), in a way that is problematic for an academic handbook. There should certainly be room for bold statements in a book which aims to push the boundaries of how we think about animal welfare, but not at the expense of recognising alternative standpoints. Also, an academic handbook is expected to be based on systematically collected evidence, so a chapter where one-third of the reference list are publications from a political lobbying group is definitely problematic.

Taking the usefulness of the handbook to its readership into account, I think more illustrations would have been helpful, in particular to give a more comprehensive picture of the situations in which animals are faring more or less well. There is also room for much more cross-referencing between chapters. These as well as the less-balanced chapters are issues that I encourage the editors to address in future editions of the handbook.

The *Routledge Handbook of Animal Welfare* fills a gap in the existing animal welfare literature, and with a few exceptions does it well. At over £200 the hardback edition is really only accessible to a minority of its potential readership, but individual chapters are open access online.

